# Barriers to Advance Care Planning in Cancer, Heart Failure and Dementia Patients: A Focus Group Study on General Practitioners' Views and Experiences

**DOI:** 10.1371/journal.pone.0084905

**Published:** 2014-01-21

**Authors:** Aline De Vleminck, Koen Pardon, Kim Beernaert, Reginald Deschepper, Dirk Houttekier, Chantal Van Audenhove, Luc Deliens, Robert Vander Stichele

**Affiliations:** 1 End-of-Life Care Research group, Ghent University & Vrije Universiteit Brussel (VUB), Brussels, Belgium; 2 LUCAS (Center for Care Research and Consultancy), Catholic University of Louvain, Louvain, Belgium; 3 Department of Public and Occupational Health, and EMGO+ Institute for Health and Care Research, VU University Medical Centre, Amsterdam, The Netherlands; 4 Heymans Institute of Pharmacology, Ghent University, Ghent, Belgium; KEMRI-Wellcome Trust Research Progrmame, Kenya

## Abstract

**Background:**

The long-term and often lifelong relationship of general practitioners (GPs) with their patients is considered to make them the ideal initiators of advance care planning (ACP). However, in general the incidence of ACP discussions is low and ACP seems to occur more often for cancer patients than for those with dementia or heart failure.

**Objective:**

To identify the barriers, from GPs' perspective, to initiating ACP and to gain insight into any differences in barriers between the trajectories of patients with cancer, heart failure and dementia.

**Method:**

Five focus groups were held with GPs (n = 36) in Flanders, Belgium. The focus group discussions were transcribed verbatim and analyzed using the method of constant comparative analysis.

**Results:**

Three types of barriers were distinguished: barriers relating to the GP, to the patient and family and to the health care system. In cancer patients, a GP's lack of knowledge about treatment options and the lack of structural collaboration between the GP and specialist were expressed as barriers. Barriers that occured more often with heart failure and dementia were the lack of GP familiarity with the terminal phase, the lack of key moments to initiate ACP, the patient's lack of awareness of their diagnosis and prognosis and the fact that patients did not often initiate such discussions themselves. The future lack of decision-making capacity of dementia patients was reported by the GPs as a specific barrier for the initiation of ACP.

**Conclusion:**

The results of our study contribute to a better understanding of the factors hindering GPs in initiating ACP. Multiple barriers need to be overcome, of which many can be addressed through the development of practical guidelines and educational interventions.

## Introduction

The expected increase in numbers of people developing dementia, the growing number of old people suffering and dying from serious chronic diseases and the rising costs of health care as a result of an aging population have focused attention on advance care planning [1]. Advance care planning (ACP) is the voluntary process by which patients discuss their future treatment and end-of-life care preferences with their care providers in case they lose the capacity to make decisions or communicate their wishes in the future. If a patient chooses to, the contents of such a discussion can be placed on record in the form of an advance statement (of wishes and preferences), or an advance decision to refuse treatment in specific circumstances and may include the appointment of a proxy decision-maker or lasting power of attorney [2], [3].

The long-term relationship between general practitioners (GPs) and their patients is considered an ideal context for introducing the subject and starting the process of ACP before the patient becomes seriously ill [4]–[6]. Evidence shows that patients are comfortable discussing ACP with their GP when their condition is stable in anticipation of future ill-health [7]. In Belgium, as in many European countries, GPs have mostly built up long-term relationships with their patients [8]. However, a cross-national retrospective study showed that, in a population of patients who died non-suddenly, GP-patient discussion of treatment preferences occurred for 25% of patients in Belgium [9]. Cancer patients are also more often involved in the process of ACP than non-cancer patients, suggesting that initiation of ACP with other patient groups has its own challenges [10].

Current international guidelines suggest that all patients with a chronic life-limiting illness should be offered ACP before time-critical situations occur [3], [11], but discussions about end-of-life care often takes place with those who are terminally ill and are relatively close to death [12]–[14]. However, an understanding of the three main illness trajectories of patients with chronic life-limiting diseases indicate that these patients may benefit from the timely initiation of ACP [15], [16]. The first trajectory is typified by cancer and generally follows a relatively predictable end-of-life course with a maintenance of good function until a rapid decline in clinical status in the last weeks of life. Heart failure is typical of the second trajectory, marked by a slow decline that is interrupted by acute deteriorations any of which might end in sudden death. For these individuals there is considerable uncertainty about when death is likely to occur. The third trajectory, typically seen in patients suffering from dementia, follows a long term period of progressive decline in functional and mental capacity before death [17]. When patients are hospitalized for health crises resulting from their chronic incurable disease, the patient may be close to death, yet there often is no clearly recognizable starting point between being very ill and actually dying [18]. Reserving ACP discussions for the end-of-life may thus deny patients the chance to adequately prepare for and plan their future care while having the decision-making capacity to do so [19].

Previous qualitative studies conducted in the UK and Australia identified a lack of time, a desire to maintain hope, prognostic uncertainty and the belief that patients are not willing or able to face discussions around death and dying as barriers to initiating ACP in primary care [20]–[22]. However, most of these studies have been focused on single patient groups (e.g. cancer patients) or on the initiation of ACP with terminally ill patients. The aim of this study is to identify the barriers, from GPs' perspective, to initiating ACP and to gain insight into any differences in barriers between the trajectories of patients with cancer, heart failure and dementia.

## Methods

### Research design

This exploratory study used the qualitative methodology of focus groups. The focus group approach was chosen because it is flexible in that it allows for open discussion and interaction in order to obtain in-depth insight into the range of views and experiences of GPs regarding barriers to initiating ACP [23].

### Recruitment of participants

Five focus group interviews with GPs were held in Flanders (Belgium) during March 2012. The participants were purposefully sampled by using several recruitment strategies in order to maximize the variation in their experience, age and practice location. Three focus groups were organized within local peer-review GP groups by contacting the chairs of six of these groups. Local peer-review GP groups are geographically determined groups of GPs from both individual and group practices that meet four times a year to discuss their practice. Every GP who wants to be accredited in Belgium, needs to be affiliated to a peer-review group and is obliged to attend two out of four meetings per year. A report from 2005 showed that more than 90% of active GPs in Belgium are affiliated to a peer-review group [24]. We chose to recruit via local peer-review groups to obtain a sample of GPs representing a wide range of experience related to the topic (maximum variation sampling) [25]. Secondly, because research shows that advance care planning usually takes place with patients who are terminally ill and close to death [26], we specifically also wanted to enroll GPs who have experience with palliative patients and communication in the last phase of life. We contacted coordinators of the palliative care networks in Flanders with the request to disseminate our invitation to GPs active in palliative home care teams. Palliative home care teams consist of experts in palliative care (physicians, nurses, psychologists) who, in addition to their own practice, advise and support palliative patients in their last phase of life and work closely with the surrounding caregivers to organize optimal care for the patient. However, because not many GPs from the palliative home care teams responded to our invitation (n = 2) we complemented this focus group with GPs not working in a palliative home care team. These other GPs were recruited through professional contacts of the palliative care coordinators that referred us to these participants (snowball sampling). A fifth focus group was organized with members from a group practice that is located in an urban region as opposed to the rural and semirural regions where the other focus groups took place.

### Data collection

**Figure 1:**
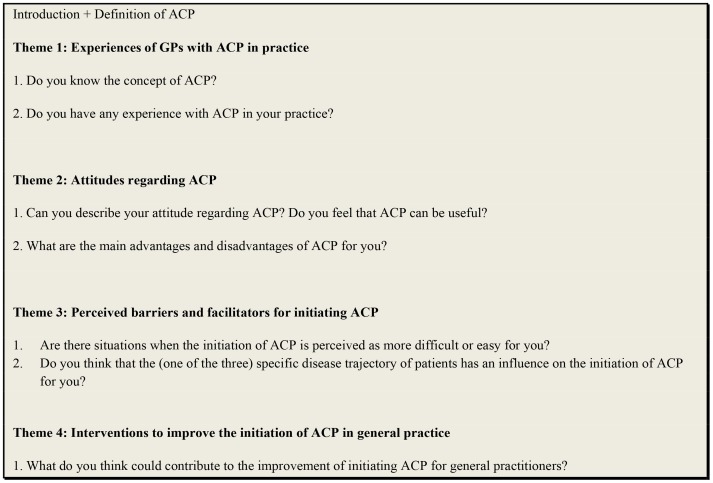
Topic guide of the focus groups with general practitioners.

A topic guide, consisting of open questions and a set of prompts for each question, was developed and reviewed within a multidisciplinary research team of sociologists (ADV, DH, LD), psychologists (KP, KB, CVA), a GP (RVS) and an anthropologist (RD), and covered four general themes: (1) experiences of GPs with ACP in their current practice (2) attitudes regarding ACP (3) perceived barriers to and facilitators for initiating ACP and (4) possible interventions to improve initiation of ACP in general practice (Figure 1). A definition of ACP was introduced at the beginning of each focus group and participants were asked whether they were familiar with this definition and the term ‘ACP’. ACP was defined as a voluntary process by which patients discuss their future treatment and end-of-life care preferences with their care providers in case they lose the capacity to make decisions or communicate their wishes for the future [2], [3].

At the start of each focus group, the participants were informed about some important ‘ground rules’ of a focus group discussion, e.g. no talking across each other, keeping the information discussed confidential, etc. Each focus group was moderated and observed by two researchers (ADV, KP, RVS or LD) and took place in a quiet meeting room. The focus groups were conducted in Flemish and were translated by the first author. The focus groups lasted on average one and a half hours and were audiotaped, for which all participants gave their informed consent. Before the interview the participants also filled in a short questionnaire regarding their own characteristics. After conducting the first two focus groups, that focused on discussing the differences between the initiating of ACP with patients with cancer, dementia and heart failure, the research team decided to explore these differences further by focusing on one of the specific patients groups in each of the three following focus groups. To improve data collection the topic guide was slightly modified after the first two focus groups, without compromising consistency. However, during these focus groups the participants drew comparisons themselves between the trajectory chosen for discussion and the other trajectories. After five focus groups, the researchers evaluated that saturation had been reached.

### Data analysis

The focus group discussions were transcribed verbatim. For analyzing the data, constant comparative analysis was used [27], [28]. Firstly, two researchers (ADV & KB) independently read and coded two full focus group transcripts. The codes were discussed and mutually compared for similarities and differences until a primary coding framework was constructed. Subsequently, the five focus group transcripts were independently read and compared with the primary coding framework by all the members of the research team. Codes were added, modified or merged where necessary. Notes were taken about the decisions that had been made during the coding process to ensure consistency of results. ADV coded the remaining transcripts by applying the final coding framework, which was additionally checked by KB and KP for agreement on interpretation. Once coding was completed, ADV & KP revised the transcripts and the coding framework. An ongoing refinement of the coding framework, by grouping the codes that had common elements, eventually resulted in categories that related to the research questions. Finally, quotes were selected (ADV & KP) and approved by the research team to illustrate the results. The qualitative analysis software QSR NVIVO 10 was used for this research.

### Ethical aspects

The research protocol was approved by the Commission of Medical Ethics of the University Hospital of Brussels. A signed informed consent was obtained from each participant before the focus group interview. Anonymity was assured by removing participant information that could lead to identification from the transcripts.

## Results

A total of 36 GPs (n = 9, n = 11, n = 4, n = 5, n = 7) attended one of the five focus groups. Participants' characteristics are presented in Table 1.

**Table 1:** Characteristics of participating GPs (N = 36).

Characteristics	FG 1 (n = 9)	FG 2 (n = 11)	FG 3 (n = 4)	FG 4 (n = 5)	FG 5 (n = 7)	Total
**Sex**						
Male	5	7	4	5	6	27
Female	4	4	0	0	1	9
**Age (years)**						
≤29	1	0	0	0	0	1
30–39	1	2	0	1	1	5
40–49	5	3	1	2	2	13
50–59	1	5	1	1	1	9
60–69	1	1	2	1	3	8
≥70	0	0	0	0	0	0
**Practice location**						
Urban	9	0	0	0	0	9
(Semi-)Rural	0	11	4	5	7	27
**Number of terminal patients in their practice in the last year**						
None	2	1	1	0	0	4
1–3	3	3	1	2	1	10
4–6	3	1	2	2	3	11
7–9	0	1	0	0	1	2
≥10	1	5	0	1	2	9
**Active in a palliative home care team**						
Yes	0	0	0	0	2	2
No	9	11	4	5	5	34
**Clinical work experience (years)**						
1–9	2	2	0	0	0	4
10–19	2	1	0	2	2	7
20–29	3	4	2	1	2	12
≥30	2	4	2	2	3	13

Although the GPs identified end-of-life care conversations as an important aspect of general practice, many of them were not familiar with the term ACP. Once a general definition was introduced, most GPs indicated that they had some experience with end-of-life care discussions but stated that they were mostly conducted in an informal way.

*It [ACP] does not have to be anything formal, it may also be just a little chat in response to… I think it is very important that that is monitored. That [ACP] does not become a formality, a consensus on paper with a hierarchical structure and a number of conditions which must be complied with. (Female GP, 40 years, FG 1)*

*Is ACP also not something that is often discussed between the lines? (Female GP, 41 years, FG 1)*

GPs with more experience and expertise in palliative care were generally more familiar with the concept and process of ACP. Positive experiences with previous ACP discussions was, according to most GPs, also considered to be an important facilitator for the initiation of ACP.

### Barriers to initiating ACP according to GPs

An overview of all the barriers that were mentioned in the focus groups is presented in Table 2. The perceived barriers were interpreted as barriers relating to the GP, to the patient and family and to the health care system (e.g. lack of time to discuss ACP in general practice). Most of the barriers identified related to the GP and could be classified as lack of GP communication skills, lack of GP knowledge regarding illness trajectories or GP attitudes and beliefs regarding ACP. There were barriers for which no differences between the trajectories of cancer, heart failure and dementia were perceived, e.g. lack of time. The barriers for which differences were indicated between these trajectories are explored in depth below.

**Table 2:** Barriers according to GPs to initiate ACP.

Barriers related to the GP
*Lack of communication & interpersonal skills of GPs:*
Difficulties for the GP with addressing non-specific patient issues
GPs not feeling comfortable in talking about death and dying
Lack of GP education about ACP
Lack of GP experience with ACP
Lack of GP experience with palliative patients
*Lack of GP knowledge regarding illness trajectories in order to initiate ACP:*
Lack of GP knowledge about treatments options in order to discuss ACP
Lack of GP familiarity with the terminal phase of illness
Difficulties for the GP with making accurate predictions of life expectancy
Difficulties for the GP to define key moments to timely initiate ACP
Lack of GP knowledge about the legal status of advance directives
*GP attitudes & beliefs concerning ACP:*
Fear of legal proceedings by implementing ADs
Fear of losing the patient as a client by discussing end-of-life care
Fear of destroying hope in the patient by initiating ACP
Fear of creating anxiety by initiating ACP
Uncertainty over appropriateness of ACP for non-chronically ill patients
Lack of trust in the value of ACP to comply with patient wishes at the end of life
Believing that patients will initiate ACP themselves if they are ready to discuss it
Believing patients do not like to discuss end-of-life care

Barriers related to the patient and family
Lack of patient initiation
Patients changing end-of-life care preferences
Patients' unawareness about the diagnosis and prognosis
Patients' and/or family members' denial about imminent death
Patients misinterpreting disease information
Future lack of decision-making capacity
Patients' family hindering end-of-life care discussions
Lack of a longstanding relationship with the patient

Barriers related to the health care system
Lack of structural collaboration with secondary care or other health care professionals
Lack of time to discuss ACP in general practice

### Differences in barriers to initiating ACP between cancer, heart failure and dementia (Table 3)

#### Lack of GP knowledge about cancer treatment options

Several GPs reported a lack of knowledge about existing treatments for different cancer types and their possible effects as a barrier, which they considered essential to the discussion of treatment decisions and end-of-life care preferences.

**Table 3:** Differences in barriers to initiate ACP between the trajectories patients with cancer, heart failure and dementia according to the GP.

	Cancer	Heart failure	Dementia	Mentioned in FG
**Barriers related to the GP:**				
Lack of GP knowledge about treatment options	↑			4, 5
Lack of GP familiarity with the terminal phase of illness		↑	↑	3, 4
Difficulties for the GP to define key moments to timely initiate ACP		↑	↑	2, 3, 4, 5
**Barriers related to the patient and family:**				
Patients' unawareness about diagnosis and prognosis		↑	↑	3, 4, 5
Future lack of decision-making capacity			↑	1, 3, 5
Lack of patient initiation		↑	↑	1, 2, 3, 4, 5
**Barriers related to the health care system:**				
Lack of structural collaboration between primary and secondary care	↑			1, 2, 3, 4, 5

↑: barrier according to GPs for the initiation of ACP with a specific patient group in comparison to the other patient groups.

*For a good planning you need to be well informed, you need to know what are the possibilities and what aren't the possibilities and I do feel that… that I often know too little. That might be because… maybe I should inquire about it more often, that may well be so, but I often get the feeling: I can't assess this anymore, what benefits does it have, or doesn't it have. I find it hard. (Female GP, 60 years, FG 5)*

This problem existed far less with regard to heart failure and dementia because of the perception by the GPs of the limited treatment options they offer.

*Heart failure is a pretty aggressive condition. Once you've got it, you can't do anything about it anymore. (Male GP, 60 years, FG 4)*

#### Lack of GP familiarity with the terminal phases of heart failure and dementia

During the focus groups, it became clear that several GPs were less familiar with the terminal phases of heart failure and dementia; not only is the terminal phase less clear than with cancer but for some GPs the life-limiting nature of heart failure and especially dementia is not always apparent. Consequently, for some GPs the recognition of the need to discuss end-of-life care does not always arise.

*Do you die from dementia? (Male GP, 58 years, FG 4)*

*Yeah, but in that case it's from age? Well yeah, you have to die once. I mean… (Male GP, 45 years, FG 4)*

However, during the focus groups, some GPs came to recognize the importance of timely initiation of ACP for heart failure and dementia patients:

*Yes, [for cancer patients] you can feel or sense that better as a GP. But for heart failure… I myself am actually a little stunned. It is true, there should actually be a discussion about it. (Male GP, FG 4, 38 years)*

*On the other hand, for a patient that is becoming demented, you may have to provide care for more than 5 years, without him knowing. So actually it is important to know what they really care about and what not. (Female GP, 35 years, FG 2)*

#### Lack of key moments for the initiating of ACP in the trajectories of heart failure and dementia

Although a number of key moments suitable for initiating ACP with cancer patients were raised during the focus groups (at diagnosis, when patients experience negative effects from medical treatment, when treatment is withdrawn and when patients are deteriorating at the end of life), the point when curative treatment is exhausted was considered the most appropriate according to most GPs. The majority of GPs acknowledged that the stage of advanced illness was too late to initiate ACP with heart failure patients but end-of-life care issues were generally raised when the patient's condition was obviously declining after numerous acute hospital admissions. For dementia patients, no key moments were identified for the initiation of ACP.

*Because it's obvious, they [cancer patients] have been diagnosed, for the time being I'm assuming most people have been informed of this diagnosis as well, and otherwise I'll deliver it myself. And so I think it's way easier to, sooner or later, start a conversation in response to that diagnosis, while it's a lot harder in case of dementia, because there's already some cognitive impairment. And it's also very difficult in case of organ failure huh, because those people are doing well, there's no sudden diagnosis, they've already been hospitalized for this before… and a patient with organ failure does get worse, but there isn't always a facilitating moment. Therefore it's not always easy to [talk to] such a person, who despite having for example lung problems, doesn't always realize he's just as terminal as a cancer patient. And I find that much harder, … (Male GP, 42 years, FG 5)*

Only a few GPs with considerable experience in end-of-life care believed that ACP should be initiated as early as possible:

*I start [ACP] with my patients as early as possible. Even if they come to me on a consultation for the first time and I see the opportunity to bring it up, I will. Because I like to know those things before it happens. I like people to give their advice before anything happens to them. It also allows for a more open and free discussion. So for me it is: the earlier, the better. And I mean, I do not snub people and ask them “what's your name and what is your vision on the end-of-life?”. But still, if an opportunity arises, I will grab it as soon as possible. I like to know how the patients sees things. (Female GP, 35 years, FG 2)*

#### A patient's lack of awareness of diagnosis and prognosis in heart failure and dementia was expressed as a barrier to the initiation of ACP compared with cancer patients

Although most heart failure patients are informed that they are suffering from a heart condition, the prognosis is not always communicated because GPs can have difficulty in explaining potential events such as the risk of sudden death without creating anxiety. The fear of creating anxiety or depression for dementia patients by explaining the expected deterioration of their mental capacity was similarly mentioned as a barrier. GPs considered it too difficult to initiate ACP at any point with patients who are unaware of their diagnosis or prognosis.

*I think we've got quite a lot of patients with dementia, but you won't immediately start telling these people: soon you won't know what you're doing anymore. It's time that you do something about it, that you start planning this, I think it's a bit of a taboo to start discussing this, to tell someone with Alzheimer's, in a year you won't know what you're doing anymore. (Male GP, 44 years, FG 3)*

According to the GPs in our focus group, cancer patients were more aware of their diagnosis as opposed to patients with heart failure and dementia, which can create an opening for the discussion of prognosis and types of treatment:

*Patients are often sent home with a diagnosis. They know what is going on, but they haven't received very specific information from the specialists. They wonder: “What will happen to me? Is there really nothing they can do for me?”. (Male GP, 60 years, FG 2)*

#### Lack of patient initiation of ACP in heart failure and dementia was mentioned as a barrier in all focus groups

The GPs described cancer patients as the easiest group with whom to initiate ACP, because they spontaneously make the association with death and dying when hearing their diagnosis.

*But it's different, because in this case the question won't come from the patient that often, I think. For cancer it's known, “cancer – death”, people do make that association. While heart failure is like, yeah, he's got heart problems.(Male GP, 38 years, FG 4)*

Because patients with dementia and heart failure are often unaware of or in denial about the life-threatening nature of their disease according to most GPs, they rarely initiate ACP themselves.

*It has never happened [that a patient with dementia starts discussing ACP on his/her own initiative] (Male GP, 69 years, FG 3)*

*Cancer patients do however. (Male GP, 65 years, FG 3)*

#### Future lack of decision-making capacity of dementia patients was given great weight as a barrier to initiating ACP

Many GPs felt uncomfortable about discussing and planning end-of-life care with patients who are losing the capacity to express a change in preference or confirm their wishes at the end of life. As patient's wishes and circumstances may change over time, some GPs were also reluctant to make formal documentation of decisions expressed by dementia patients.

*Because I think for the category of people with a deteriorating level of consciousness it's a different story than for people with cancer or kidney failure, with whom you can hold conversations until the very end sometimes… I think this is a really difficult category of people compared to a person who can still talk to you about what he wants or doesn't want. (Female GP, 44 years, FG 1)*

*I think in the really early stages of dementia we actually don't discuss this with the patient because it's often diagnosed very late. At that moment, discussing it is actually no longer possible. (Male GP,44 years, FG 3)*

On the other hand, some GPs considered the future loss of a patient's decision-making capacity the very reason for initiating ACP:

*It is much better that you discuss this in advance, rather than to discuss it with the patient's family because the patients has dementia. If you previously haven't talked about it, than you actually have missed your chance. So yes, I believe that ACP discussions should start early with the patient instead of talking to the family, the nurse, or the care team around a patient that is not able to communicate anymore. (Male GP, 65 years, FG 5)*

In some focus groups, doubts about the legality of advance directives (ADs) drafted by dementia patients and anxieties about possible legal proceedings that may follow their implementation were also mentioned as a barrier to initiate ACP discussions:

*If the patient signs, and he has been diagnosed with dementia, where do you stand, is it still legal? He signed while, according to the court of justice, he no longer knew what he was doing. (Male GP, 44 years, FG 3)*

#### Lack of structural collaboration between primary and secondary care for cancer patients was mentioned in all focus groups as a barrier to initiating ACP

In the case of cancer particularly, limited contact with patients and lack of information from specialists during treatment phases were considered as factors that hinder the initiation of ACP.

*The patient used to be able to report back to you once in a while about what had or hadn't been decided. But nowadays they [the hospital] actually keep them to themselves for a very long time, even in a palliative situation I think. Well, at certain wards this is definitely the case. You don't have to draw their blood anymore, they don't have to come and talk to you. They do that at the hospital for a really long time, even the moment they say, now we can't do anything anymore, maybe you should go to the palliative unit now, while the patient actually won't hear a lot about dying at home if we don't come and explain it, I think. (Female GP, 49 years, FG 1)*

Some GPs evaluated the collaboration between GPs and specialist more positively, but acknowledged that was mostly due to a longer relationship or a past experience between the GP and specialist:

*I feel that you almost need to have a personal relationship with a specialist before you can have any impact. (Male GP, 30 years, FG 1)*

*Yes, there is indeed a big difference. I also hear that from colleagues who work in a completely different area, where there is a smaller local hospital. They have a much closer relationship with the specialist than here. I think here some older colleagues who have some experience have much more direct contact with the specialists, so a lot more things are possible. (Male GP, 29 years, FG 1)*

The tendency of specialists to persist with curative treatment even when patients are deteriorating appeared to be an obstacle to initiating ACP for many GPs.

*You do tell the family, just keep him here because he's dying, he only has a couple of weeks left, whereas the oncologist told them 2 days before ‘you really need to come, because this will still make you feel good’. So there you are then. (Male GP, 65 years, FG 5)*

*As a GP, you really can't go against this. (Male GP, 46 years, FG 5)*

## Discussion

### Summary of main findings

The GPs in our study reported multiple barriers to initiating ACP relating to their own characteristics, the characteristics of the patients and their families and the structure and organization of the health care system. They also perceived certain of these barriers to relate more to the specific trajectories of cancer, heart failure or dementia. In cancer patients, a GP's lack of knowledge about treatment options and the lack of structural collaboration between the GP and specialist were expressed as barriers. Barriers that occurred more often with heart failure and dementia patients were the lack of GP familiarity with the terminal phase, the lack of key moments to initiate ACP, the patient's lack of awareness of their diagnosis and prognosis and the fact that patients did not often initiate such discussions themselves. The future lack of decision-making capacity of dementia patients was reported by the GPs as a specific barrier for the initiation of ACP.

### Strengths and limitations of the study

To our knowledge, this is the first qualitative study providing in-depth insight into the similarities and differences in the barriers to initiation of ACP between cancer, heart failure and dementia patients, as perceived by the GP. By using different sampling strategies we gained insight into the complex range of views and experiences regarding ACP in daily practice from GPs with diverse backgrounds, experience and interest in ACP. This study provides a better understanding of the problems that need to be overcome when developing interventions or training programmes to enhance the initiation of ACP in general practice [29]. Our description of the barriers remained as close as possible to the phrasing used in the focus groups and we divided them into three main categories as relating to the GP, to the patient and family and to the health care system; however many barriers are interrelated and should not be interpreted as isolated factors. The focus group composition may have presented a limitation. Most of the participating GPs were male (n = 27), so female GPs (n = 9) were underrepresented, as were GPs younger than 39 years (n = 6 vs. n = 30). Secondly, the perspective of the GPs themselves is valuable in obtaining the information essential for making changes in education and innovation in practice. However, other perspectives such as those of patients and family members, and of specialists, could provide additional insights that could also contribute to a better understanding of the problems of ACP and to the formation of useful educational approaches.

### Comparison with existing literature

The findings of this qualitative study confirm many barriers to GP initiation of ACP found in previous studies [1], [5], [30], but are here placed in the context of the three illness trajectories of cancer, heart failure or dementia. Four of the seven barriers for which a difference was identified, pertained to heart failure and dementia patients. The barriers pertaining to both heart failure and dementia patients seem mainly to be consequences of the less predictable disease course of these conditions, leading to GPs experiencing difficulty with predicting the terminal phase of disease [31], [32]. The clearer demarcation between curative and palliative care in cancer patients, often used as a trigger and also considered as the most appropriate moment by the GPs in our focus groups to initiate ACP, is less distinct in heart failure and dementia. This challenges GPs to identify other key moments to initiate ACP, one of the main problems for GPs that needs to be addressed according to previous studies [33], [34]. Previous research also shows that GPs find diagnosing heart failure and giving a prognosis particularly challenging, making it difficult for them to relay information back to patients [31]. Many patients are never actually told that they have heart failure because doctors are reluctant to use the term [35] and similar concerns have been raised for dementia [36]. Communicating a diagnosis and prognosis is however an important element in informing patients of treatment and end-of-life care choices. Recently, experiential skills-building communication training in cancer has been shown to improve clinicians' skills in communication about end-of-life care [37], [38]. As GPs' experience with ACP was also considered to be a facilitator in this study, offering GPs such training may improve their confidence and skills with initiating ACP for all patients in relation to chronic life-limiting conditions.

The barriers pertaining to cancer patients (lack of knowledge about treatment options and lack of structural collaboration between primary and secondary care) seem to be related to the increasing specialization and complexity of cancer treatments. While heart failure and dementia are largely managed in primary care, most follow-up and surveillance of cancer patients remains in the hands of specialists [39]. As a consequence, GPs often lose touch with their patients during active treatment, which is not countered by effective collaboration or information transmission between GPs and specialists [40]. Although GPs are generally identified, including by specialists, as the most appropriate professionals to initiate ACP, GPs themselves have reported a lack of clarity about whose role it is [41]. Addressing a GP's need for detailed and timely information regarding their patient's care by improving the standard communication procedures between GPs and specialists could facilitate the initiation of ACP for GPs. It is however also important for GPs to acknowledge that the discussion of treatment options is only a part of ACP and the difficult subject of end-of-life care should not be disregarded.

The future lack of decision-making capacity was the only specific barrier reported to the initiation of ACP with dementia patients. Previous research has pointed out that diagnosing dementia is a complex task and usually when diagnoses are formally assessed, patients are already suffering from some form of cognitive impairment [36], [42]. However, in the early stage of dementia there is a time span when patients can talk about their values and goals in a way that could inform end-of-life care decisions when they have lost the capacity to make decisions [43]. A number of explanations are possible for the reservations GPs express about assessing such patients' capacity to participate in ACP discussion. Firstly, as this study also shows, GPs have doubts about the relevance or the value of ACP in the context of future loss of capacity to confirm previously planned decisions and they perhaps adopt the attitude that advance care planning for dementia patients is invalid [44]. Secondly, physicians don't always recognize that dementia can be a terminal illness, which may explain why patients with dementia are less likely than those with cancer to have advance directives [45]. Finally, a lack of knowledge about the extent to which advance statements or decisions should be followed and to which extent they are legally binding, which was reported as a barrier by the GPs in our study, may further strengthen their negative attitudes or beliefs.

### Conclusion and implications for practice, policy and research

Because GPs in Belgium, as in many other countries, have a central role in the coordination of patients' care, they are considered to be ideally placed to initiate ACP with their patients. To put this into practice, a broad range of barriers relating to the GPs, to the patients and family and to the health care system needs to be overcome. Educational training and the development of guidelines adapted to the Belgian context can play an important role in achieving this goal [46], as most of the perceived barriers identified in this study were related to skills, knowledge and attitudes. Future large scales studies may contribute to a more complete picture of the prevalence and importance of the barriers encountered by GPs when initiating ACP. Future research is also necessary on when GPs can elicit patients' wishes for future end-of-life care before time-critical situations occur, especially for patients with heart failure and dementia (two conditions with a less predictable end-of-life trajectory than cancer patients [47]). Introducing the concept of ACP in advance of illness and as part of standard care may be a realistic strategy and requires further research.
